# A Region-Aware Structured Framework Improves Prediction of Gene Expression from DNA Methylation

**DOI:** 10.34133/csbj.0138

**Published:** 2026-06-26

**Authors:** Zhixing Zhong, Jinglu Hu

**Affiliations:** Graduate School of Information, Production and Systems, Waseda University, Fukuoka 808-0135, Japan.

## Abstract

DNA methylation is a key epigenetic modification that plays an important role in gene expression regulation and disease development. Inferring gene expression from DNA methylation provides a computational strategy for cross-omics integration and facilitates the exploration of regulatory relationships between epigenetic modifications and transcription. However, the regulatory effects of methylation on gene expression often exhibit complex characteristics, and methylation in different functional regions of a gene may follow distinct regulatory patterns. Existing methods typically lack the capacity to model such region-aware nonlinear relationships. In this study, we propose RSMethy-Net, a neural network framework based on region-aware encoding for predicting gene expression from DNA methylation data. The model incorporates grouped region encoding modules for different gene functional regions to capture their latent regulatory patterns and characterize methylation–expression associations under a nonlinear predictive framework. We systematically evaluated RSMethy-Net across 6 cancer cohorts. Experimental results demonstrate that RSMethy-Net outperforms multiple baseline methods in predictive performance. Furthermore, by integrating region design with model interpretability analyses, the framework can quantify the contributions of different gene regions to predictions, providing insight into methylation–expression associations under a nonlinear predictive setting.

## Introduction

In recent years, the rapid development of omics technologies has enabled researchers to systematically characterize molecular features of biological processes at multiple scales, significantly advancing our understanding of underlying molecular mechanisms [1,2]. Among these, DNA methylation, as a key epigenetic modification, has attracted widespread attention and is considered to play important roles in disease progression, transcriptional regulation, and cell differentiation [3,4]. Numerous studies have reported that aberrant methylation patterns are closely associated with various diseases [4,5]. For example, in cancer, aberrant methylation has been associated with the silencing of tumor suppressor genes and the activation of oncogenes, thereby contributing to cancer progression [6]. These findings suggest that DNA methylation participates in disease-related regulatory processes by influencing gene expression. Therefore, DNA methylation not only serves as an important biomarker for disease but also acts as a critical regulatory bridge between the epigenetic and transcriptional layers.

In the context of multi-omics research, the joint profiling of DNA methylation and transcriptomic data provides valuable insights into epigenetic gene regulatory mechanisms across multiple molecular layers [7]. However, in practice, sample-level matched multi-omics datasets remain relatively limited, which restricts the broader application.

Computational approaches that infer information in one omics layer from another have gradually emerged as an important direction in multi-omics research [8]. In particular, recent studies have introduced machine learning and deep learning frameworks for cross-omics translation tasks, including representative methods such as BABEL [9], scMOG [10], and MultiVI [11]. These approaches have demonstrated promising performance in modeling relationships between molecular modalities. However, existing epigenomics-based cross-modal methods have primarily focused on chromatin accessibility modalities such as Assay for Transposase-Accessible Chromatin using sequencing (ATAC-seq), while studies based on DNA methylation remain relatively limited. Predicting gene expression from DNA methylation not only provides complementary transcriptomic information when matched expression data are unavailable, but also offers a useful framework for investigating regulatory relationships between methylation and gene expression.

Machine learning methods based on DNA methylation data have been applied to various tasks [12,13], suggesting their potential for gene expression prediction. Several studies have specifically explored predicting gene expression from DNA methylation using machine learning models, including the linear regression-based geneEXPLORER [14] and the convolutional neural networks (CNNs)-based DeepMethyGene [15]. However, the regulatory relationship between DNA methylation and gene expression is not entirely linear. Linear models may have limitations in capturing complex regulatory relationships and may fail to identify potential nonlinear patterns. On the other hand, CNNs have demonstrated strong capabilities in modeling signals with local continuous structures and have achieved remarkable performance in tasks such as DNA sequence analysis [16,17]. However, unlike continuous DNA sequences, methylation probes are unevenly distributed along the genome [18], which may limit the effectiveness of convolution-based methods for modeling methylation data.

Moreover, accumulating evidence suggests that the relationship between DNA methylation and gene expression varies across different genomic functional regions. For example, methylation near transcription start sites (TSS) is often negatively associated with gene expression levels [19–21], whereas methylation within gene body regions is frequently associated with higher gene expression levels [22–24]. However, existing approaches often treat methylation features from different genomic regions as a unified input to the model [25–27], lacking explicit modeling of region-specific regulatory differences. In addition, while recent deep learning-based studies on DNA methylation have increasingly focused on improving interpretability [28], region-level interpretability in gene expression prediction tasks from DNA methylation remains relatively limited.

Therefore, we propose RSMethy-Net, a region-aware neural network framework for predicting gene expression from DNA methylation data. Different from approaches that treat methylation profiles as a unified input or rely on generic convolutional architectures, we introduce a region-aware encoding strategy based on genomic functional annotations. This design is motivated by the heterogeneous regulatory roles of DNA methylation across different genomic regions, which may not be fully captured by global representations alone. Furthermore, RSMethy-Net provides interpretable region-level contributions, allowing functional genomic regions to be quantitatively assessed in the prediction process.

We conducted a comprehensive evaluation of the proposed method on 6 cancer datasets from The Cancer Genome Atlas (TCGA) project [29]. Experimental results demonstrate that the proposed region-aware encoding neural network framework consistently improves prediction performance across multiple cancer types. In addition, the interpretability analysis provides region-level insights into the contributions of methylation signals to model predictions. Overall, this study provides a new methodological framework for computational cross-omics integration.

## Materials and Methods

### Datasets and preprocessing

DNA methylation and gene expression data used in this study were obtained from the TCGA project [29] and downloaded via the UCSC Xena platform [30]. We analyzed matched multi-omics data from 6 cancer types, including head and neck squamous cell carcinoma (HNSC), lung adenocarcinoma (LUAD), stomach adenocarcinoma (STAD), colon adenocarcinoma (COAD), uterine corpus endometrial carcinoma (UCEC), and pancreatic adenocarcinoma (PAAD). DNA methylation data were profiled using the Illumina HumanMethylation450 BeadChip platform, and gene expression data were obtained from the RNA-seq HiSeq V2 expression matrix.

Data preprocessing and filtering were performed following procedures described in a previous study [14]. The original methylation levels were represented as *β* values and were transformed into *M* values for subsequent analysis. The missing rate of each cytosine-phosphate-guanine (CpG) probe was then calculated, and probes with more than 20% missing values were removed. Missing values were imputed using the grooMethy function implemented in the REMP package [31]. For gene expression data, lowly expressed genes with an average expression value ≤1 were filtered out, where expression values were computed from RSEM-normalized counts and log2-transformed as log2(RSEM + 1). Only samples that were present in both the methylation and expression datasets were retained. Following the previous study [14], CpG probes located within ±10 Mb of each gene were selected as candidate methylation features. At the gene level, only genes whose promoter regions contained at least one CpG probe were kept for analysis. Promoter regions were defined based on the hg19 genome annotation, and the genomic overlap between CpG probes and promoter regions was calculated using the GenomicRanges package [32]. Details of filtering criteria and CpG window size selection are provided in Note S1 and Figs. S1 and S2.

After preprocessing and filtering, the numbers of samples and genes included for analysis in each cancer cohort are summarized in Table S1.

### Model architecture and experimental setup

**Fig. 1.**
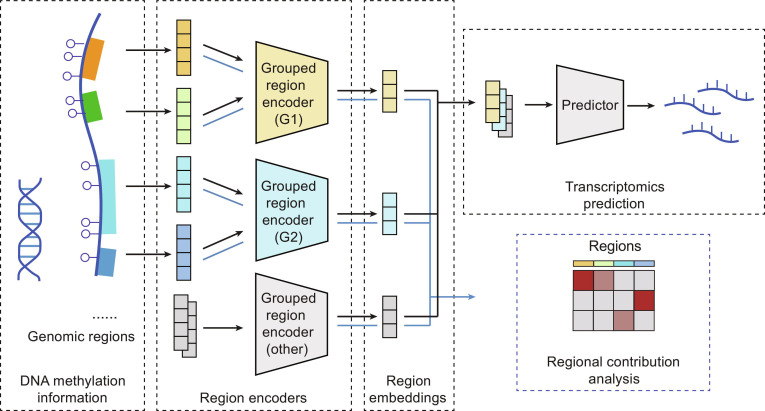
Overview of RSMethy-Net. RSMethy-Net is a region-aware encoding neural network framework for predicting gene expression from DNA methylation data. Methylation features are first organized according to genomic regions (DNA methylation information), with regions exhibiting consistent regulatory tendencies further grouped and processed by independent grouped region encoders (Region encoders) to learn regional embeddings (Region embeddings). These embeddings are then integrated to generate the final transcriptomic prediction (Transcriptomics prediction). Gradient-based attribution offers insight into regional contribution patterns, supporting interpretability (Regional contribution analysis).

RSMethy-Net is a neural network framework based on region-aware encoding, designed to predict gene expression levels from DNA methylation profiles. The overall architecture of the framework is illustrated in Fig. 1. The model uses CpG methylation levels as input features, while gene expression levels serve as prediction targets. It is trained end-to-end to learn the regression mapping from methylation profiles to gene expression levels.

To characterize the differential regulatory relationships of DNA methylation across genomic regions, methylation sites were first partitioned into multiple functional regions according to genomic annotation information. Specifically, 6 commonly used genomic regions associated with gene regulation were considered, including TSS1500 and TSS200 (upstream promoter regions involved in transcription initiation), 5′UTR (5′ untranslated region related to transcriptional and translational regulation), 1stExon (first exon region), Body (gene body region), and 3′UTR (3′ untranslated region associated with posttranscriptional regulation). These region definitions are derived from the Illumina HumanMethylation450 BeadChip platform annotation.

Subsequently, regions with similar methylation–expression association patterns were grouped together and jointly encoded. Specifically, genomic regions were divided into 3 groups: G1, including TSS1500, TSS200, 1stExon, and 5′UTR, which are generally associated with negative methylation–expression relationships; G2, including Body and 3′UTR, which are more frequently associated with positive methylation–expression relationships; and an additional “others” group containing probes outside these annotated regions. This design enables the model to capture shared regulatory patterns while preserving regional biological characteristics. Further evaluation of the regional grouping strategy is provided in Note S2 and Table S2.

Specifically, for a target gene ℊ, let the set of associated CpG probes be defined as:

$$P_{ℊ}=\left\{p_{1},\;p_{2},\;\ldots ,\;p_{P}\right\}$$ (1)

where P denotes the total number of CpG sites associated with the gene. According to the methylation probe annotation, each CpG probe can be mapped to a corresponding genomic functional region. We therefore define a mapping function:

$$\phi :P_{ℊ}\to R$$ (2)

where R denotes the set of genomic regions, including TSS200, TSS1500, 5′UTR, 1stExon, Body, and 3′UTR, with an additional category “others” for probes that do not fall into these regions.

$$\begin{array}{c} \begin{array}{c} \begin{array}{c} R=\left\{\;\mathrm{TSS}200,\;\mathrm{TSS}1500,\;5^{\prime }\mathrm{UTR},\;1\text{stExon},\;\text{Body},\;3^{\prime }\mathrm{UTR},\;\text{others}\;\right\} \end{array} \end{array} \end{array}$$ (3)

According to this mapping relationship, the subset of probes corresponding to each region can be obtained, denoted as $P_{ℊ}^{\left(r\right)}$:

$$P_{ℊ}^{\left(r\right)}=\left\{p_{i}\in P_{ℊ}|\phi \left(p_{i}\right)=r\right\}$$ (4)

Regions with similar methylation–expression association patterns were further grouped into higher-level regional categories. Specifically, regions that are generally linked to transcriptional repression under increased methylation levels were grouped as $G_{1}$, whereas regions more frequently exhibiting positive methylation–expression associations were grouped as $G_{2}$; all remaining probes were assigned to the “others” group. For notational convenience, the “others” category is denoted as $G_{3}$.

$$\begin{array}{c} G_{1}=\left\{\mathrm{TSS}1500,\;\mathrm{TSS}200,1\text{stExon},\;5^{\prime }\mathrm{UTR}\right\} \end{array}$$ (5)

$$G_{2}=\left\{\text{Body},\;3^{\prime }\mathrm{UTR}\right\}$$ (6)

$$G_{3}=\left\{\text{others}\right\}$$ (7)

Let $G_{m}$ denote the -mth grouped regional category, where $m\in \left\{1,\;2,\;3\right\}$. For each grouped category $G_{m}$, the corresponding methylation features were merged into a grouped feature vector denoted by:

$$X_{ℊ}^{\left(G_{m}\right)}\in {\mathbb{R}}^{d_{m}}$$ (8)

Here, $d_{m}$ denotes the number of probes in grouped category region $G_{m}$. Accordingly, the methylation input for gene ℊ can be represented as a collection of feature vectors across all regions:

$$Xℊ=\left\{X_{ℊ}^{\left(G_{1}\right)},\;X_{ℊ}^{\left(G_{2}\right)},\;X_{ℊ}^{\left(G_{3}\right)}\right\}$$ (9)

Prior to inputting the data into the model, the methylation features for each region were standardized. Specifically, the mean and standard deviation of each CpG probe were calculated from the training samples, and *z*-score normalization was applied. Denoting the original features as $X_{ℊ}^{\left(G_{m}\right)}$, the standardized input features are given by:

$$X_{ℊ}^{\left(\tilde{G_{m}}\right)}=\frac{X_{ℊ}^{\left(G_{m}\right)}-\mu_{m}}{\sigma_{m}}$$ (10)

Here, $\mu_{m}$ and $\sigma_{m}$ denote the mean and standard deviation of the CpG probes in category $G_{m}$ computed from the training set.

Subsequently, the standardized feature subset for each region, $X_{ℊ}^{\left(\tilde{G_{m}}\right)}$, is fed into an independent grouped region encoder for representation learning. Each grouped region encoder was implemented independently without parameter sharing across groups. Formally, each grouped region encoder can be represented as:

$$f_{m}:{\mathbb{R}}^{d_{m}}\to {\mathbb{R}}^{n_{m}\cdot k}$$ (11)

Here, $d_{m}$ denotes the number of CpG probes assigned to group $G_{m}$, $n_{m}$ denotes the number of functional regions within group $G_{m}$, and k denotes the dimensionality of each region-level embedding. In this study, we set k=64. The output is a flattened single vector of dimension $n_{m}\cdot k$. Detailed model architecture is provided in Note S3.

After the transformation by the encoder, each group produces a group-specific embedding that captures the methylation patterns within that region. The embeddings for all groups are concatenated and fed into the prediction function $F\left(\cdot \right)$ to predict the expression level of the target gene. The overall model can be formulated as:

$${\hat{y}}_{ℊ}=F\left(\overset{M}{\underset{m=1}{⨁}}f_{m}\left(X_{ℊ}^{\left(\tilde{G_{m}}\right)}\right)\right)$$ (12)

Here, ⨁ denotes the feature concatenation operation, and ${\hat{y}}_{g}$ represents the predicted expression level of the target gene ℊ. M denotes the total number of grouped regional categories. In this study, M=3.

The expression values of each target gene were standardized using the training set statistics. Let y denote the original HiSeq V2 expression value, and $\tilde{y}$ its standardized form:

$$\tilde{y}=\frac{y-\mu_{y}}{\sigma_{y}}\cdot s$$ (13)

where $\mu_{y}$ and $\sigma_{y}$ denote the mean and standard deviation of expression values in the training set, and s is a scaling factor used to bring the expression values into a numerical range suitable for neural network training.

The model was trained using a composite loss function, consisting of Smooth L1 loss [33] and a loss based on the Pearson correlation coefficient, which together are used to control prediction error and encourage consistency of expression patterns. The regression error was measured using Smooth L1 loss.

$$L_{\mathrm{reg}}=\text{SmoothL}1\left(\tilde{y},\;\hat{y}\right)$$ (14)

The correlation loss was defined as:

$$L_{\mathrm{pcc}}=1-\mathrm{PCC}\left(\tilde{y},\;\hat{y}\right)$$ (15)

The final training objective is given by:

$$L=L_{\mathrm{reg}}+\lambda L_{\mathrm{pcc}}$$ (16)

Model parameters were optimized using the Adam optimizer [34] with an initial learning rate of $1\times 10^{-4}$ and weight decay of $1\times 10^{-3}$. To prevent overfitting, early stopping was applied: training was terminated if the validation performance did not improve for 5 epochs. The maximum number of training epochs was set to 500. The model was implemented in PyTorch [35].

Smooth L1 loss was used to provide robust optimization of prediction error, while PCC loss was introduced to encourage consistency between predicted and true expression patterns. We also explored several architectural and regularization variants in an early prototype, such as partially parameter-shared encoders and additional regularization strategies, but no consistent performance improvements were observed.

Since the model is trained and predicts on standardized expression values $\tilde{y}$, the predicted results need to be transformed back to the original scale for fair comparison with observed expression values and other methods. Let $\hat{y}$ denote the model output; the corresponding prediction on the original scale, ${\hat{y}}^{\mathrm{raw}}$, is obtained as:

$${\hat{y}}^{\mathrm{raw}}=\frac{\hat{y}}{s}\sigma_{y}+\mu_{y}$$ (17)

Model performance was evaluated using ${\hat{y}}^{\mathrm{raw}}$, which was also considered the final predicted gene expression value.

### Baseline methods and ablation study

We selected multiple baseline methods for comparison, including an elastic net regression model related to geneEXPLORER, which serves as a representative linear method (hereafter referred to as LR) [14], random forest (RF) [36], support vector machine (SVM) [37], and CNNs related to DeepMethyGene [15]. To improve comparability across methods, all baseline models were reimplemented and evaluated using the same preprocessed input features, target outputs, and training-validation splits as RSMethy-Net. Performance was assessed using 5-fold cross-validation, with all models trained independently from scratch and evaluated on the corresponding validation set in each fold.

The LR baseline was reimplemented with reference to the methodology described in geneEXPLORER [14], using the glmnet R package to perform elastic net regression [38–40], a regularized linear regression method. In this study, elastic net regression was applied solely on the training set for feature selection and hyperparameter tuning (e.g., λ and α), and performance was evaluated on the validation set.

RF and SVM were implemented using Python’s scikit-learn library, with RandomForestRegressor and SVR functions to construct the RF regressor and support vector regressor [36,37,41]. To reduce redundancy and noise, the input features for SVM were transformed using principal component analysis [42].

The CNN baseline was reimplemented with reference to the architectural design described in DeepMethyGene [15]. In the experiments, CpG probes were input to the network in genomic order. A series of one-dimensional convolutional layers (1D Conv) with residual connections were used to extract features. The convolutional outputs were then flattened and passed through fully connected layers, followed by a prediction head to generate the final output.

Furthermore, to assess the contribution of region design and nonlinear modeling to performance, an ablation model without region design was constructed. This model maintained the same network scale and hyperparameters as RSMethy-Net, with a single large encoder (approximately equivalent in total size to all regional encoders combined) replacing the original region-aware encoders. All other settings and evaluation procedures were kept consistent with RSMethy-Net.

### Performance evaluation

Model performance was evaluated using several commonly used regression metrics, including the coefficient of determination ($R^{2}$), Pearson correlation coefficient (PCC), mean squared error (MSE), and mean absolute error (MAE). All metrics were computed using the model’s final predicted gene expression values and the corresponding original HiSeq V2 expression values. $R^{2}$, MSE, and MAE were calculated using r2_score, mean_squared_error, and mean_absolute_error functions from the scikit-learn library [41], while PCC was calculated using stats.pearsonr function from the scipy library [43]. To assess statistical significance of performance differences between models, paired *t* tests were performed using the stats.ttest_rel function from scipy library [35].

During 5-fold cross-validation, 80% of samples were used for training and 20% were used for validation in each fold, with each sample serving as validation data once. Cross-validation was performed independently within each cancer cohort, where samples were randomly partitioned into training and validation sets for each cancer type separately. Predicted expression values from all folds were combined to obtain predictions for the entire dataset, which were then used to calculate overall performance metrics.

### Model interpretability

Model interpretability was assessed using a gradient-based feature attribution approach [44,45], in which feature contributions were first computed at the individual CpG probe level and then aggregated according to the original genomic region annotations to evaluate region-specific importance.

Specifically, let $x_{\mathrm{ij}}$ denote the standardized methylation value of probe $p_{j}$ in sample i. Its contribution to the model prediction is defined as:

$$c_{\mathrm{ij}}=\left|x_{\mathrm{ij}}\cdot \frac{\partial \hat{y_{i}}}{\partial x_{\mathrm{ij}}}\right|$$ (18)

where $c_{\mathrm{ij}}$ reflects the sensitivity of the prediction to changes in the feature.

The probe-level contribution for probe $p_{j}$ was calculated by averaging over all n samples in the validation set:

$$C_{j}=\frac{1}{n}\sum_{i=1}^{n}c_{\mathrm{ij}}$$ (19)

where n denotes the number of samples in the validation set.

Based on the region mapping described above, each region r∈R corresponds to a subset of probes $P_{ℊ}^{\left(r\right)}$. The region-level contribution was then computed by averaging the contributions of all probes in the region:

$$C_{r}=\frac{1}{\left|P_{ℊ}^{\left(r\right)}\right|}\sum_{p_{j}\in P_{ℊ}^{\left(r\right)}}C_{j}$$ (20)

In this study, $C_{r}$ was used to quantify the contribution of region r to the prediction and served as the basis for subsequent analyses.

## Results

### Performance across cancer types

We systematically evaluated the predictive performance of RSMethy-Net across 6 TCGA cancer cohorts (HNSC, LUAD, STAD, COAD, UCEC, and PAAD) [29] and compared it with multiple baseline methods, including LR, RF, SVM, and CNNs. In addition, an ablation model without region design was constructed to assess the contribution of regional modeling while maintaining the overall network size. All methods were reimplemented and evaluated using the same preprocessing pipeline and identical training–validation splits (detailed in Materials and Methods). Model performance was assessed using *R*^2^, PCC, MSE, and MAE under 5-fold cross-validation.

**Fig. 2.**
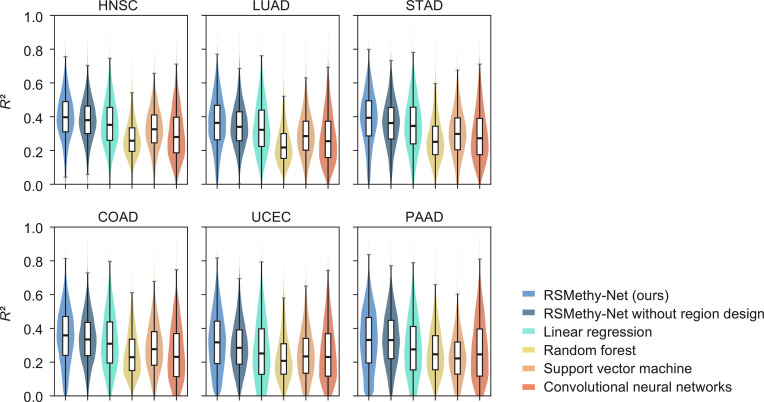
Performance comparison of RSMethy-Net and other methods across 6 The Cancer Genome Atlas (TCGA) cancer cohorts. Distribution of gene-level *R*^2^ values for all predicted genes under 5-fold cross-validation at the patient level in head and neck squamous cell carcinoma (HNSC), lung adenocarcinoma (LUAD), stomach adenocarcinoma (STAD), colon adenocarcinoma (COAD), uterine corpus endometrial carcinoma (UCEC), and pancreatic adenocarcinoma (PAAD).

The overall comparison results are summarized in Fig. 2, Table 1, Fig. S3, and Tables S3 and S4. Figure 2 shows the distribution of *R*^2^ values for all predicted genes across the 6 cancer types. The corresponding statistical tests are provided in Fig. S3. These statistical analyses are based on gene-level pairwise comparisons and assess the consistency and stability of performance improvements across genes. Table 1 summarizes the overall predictive performance for each method in the 6 cancers, reporting the median *R*^2^, PCC, MSE, and MAE across all predicted genes. Table S3 further reports the gene-level mean and standard deviation (SD) of *R*^2^ and PCC for all methods in each cancer cohort, while Table S4 summarizes the overall median performance and corresponding SD across the 6 cancer cohorts.

**Table 1.** Summary of predictive performance of 6 methods across 6 TCGA (The Cancer Genome Atlas) cancer cohorts. Median gene-level *R*^2^, PCC, MSE, and MAE under 5-fold patient-level cross-validation are reported for each cancer type. Boldface indicates the best performance for each cancer type.

Cancer type	Method	Performance			
		Median *R*^2^	Median PCC	Median MSE	Median MAE
HNSC	Convolutional neural networks	0.2800	0.5322	0.5019	0.5400
	Support vector machine	0.3255	0.5811	0.4816	0.5306
	Random forest	0.2578	0.5429	0.5307	0.5569
	Linear regression	0.3518	0.5946	0.4610	0.5192
	RSMethy-Net without region design	0.3798	0.6181	0.4468	0.5041
	**RSMethy-Net (ours)**	**0.3969**	**0.6321**	**0.4322**	**0.4977**
LUAD	Convolutional neural networks	0.2550	0.5076	0.4613	0.5190
	Support vector machine	0.2852	0.5458	0.4555	0.5155
	Random forest	0.2175	0.4989	0.4949	0.5357
	Linear regression	0.3224	0.5691	0.4216	0.4978
	RSMethy-Net without region design	0.3400	0.5848	0.4172	0.4888
	**RSMethy-Net (ours)**	**0.3636**	**0.6057**	**0.4011**	**0.4812**
STAD	Convolutional neural networks	0.2724	0.5254	0.4743	0.5310
	Support vector machine	0.2978	0.5597	0.4751	0.5300
	Random forest	0.2514	0.5269	0.5034	0.5462
	Linear regression	0.3453	0.5894	0.4337	0.5093
	RSMethy-Net without region design	0.3624	0.6039	0.4297	0.4986
	**RSMethy -Net (ours)**	**0.3936**	**0.6299**	**0.4085**	**0.4893**
COAD	Convolutional neural networks	0.2311	0.4882	0.3396	0.4501
	Support vector machine	0.2770	0.5453	0.3306	0.4434
	Random forest	0.2295	0.5090	0.3480	0.4543
	Linear regression	0.3089	0.5582	0.3142	0.4336
	RSMethy-Net without region design	0.3341	0.5811	0.3035	0.4198
	**RSMethy-Net (ours)**	**0.3586**	**0.6023**	**0.2953**	**0.4166**
UCEC	Convolutional neural networks	0.2301	0.4871	0.7014	0.6355
	Support vector machine	0.2338	0.5103	0.7282	0.6424
	Random forest	0.2076	0.4796	0.7333	0.6476
	Linear regression	0.2511	0.5061	0.6910	0.6321
	RSMethy-Net without region design	0.2848	0.5387	0.6730	0.6090
	**RSMethy-Net (ours)**	**0.3172**	**0.5695**	**0.6389**	**0.5965**
PAAD	Convolutional neural networks	0.2460	0.5036	0.3569	0.4497
	Support vector machine	0.2219	0.5346	0.3837	0.4667
	Random forest	0.2464	0.5223	0.3690	0.4521
	Linear regression	0.2754	0.5290	0.3473	0.4411
	RSMethy-Net without region design	0.3307	0.5825	0.3230	0.4200
	**RSMethy-Net (ours)**	**0.3313**	**0.5837**	**0.3212**	**0.4194**

PCC, Pearson correlation coefficient; MSE, mean squared error; MAE, mean absolute error; HNSC, head and neck squamous cell carcinoma; LUAD, lung adenocarcinoma; STAD, stomach adenocarcinoma; COAD, colon adenocarcinoma; UCEC, uterine corpus endometrial carcinoma; PAAD, pancreatic adenocarcinoma

Across all cancers, the linear model (LR) achieved moderate predictive performance, with a median *R*^2^ of 0.3092 ± 0.0395 (mean ± SD, n = 6 cancer types), indicating that linear models can capture part of the relationship between CpG methylation and gene expression. However, nonlinear modeling further improved predictive performance. RSMethy-Net achieved a median *R*^2^ of 0.3602 ± 0.0321 (mean ± SD, *n* = 6 cancer types), representing a 16.49% improvement over LR, with consistent gains observed across all 6 cancer types (Fig. 2 and Table S4). Statistical testing confirmed that the improvements of RSMethy-Net over other methods were significant in all cancers (Fig. S3).

Comparing the full model with the ablation model without region design revealed that incorporating regional modeling consistently enhanced performance across all cancer types (Fig. 2, Table 1, and Table S4). The median *R*^2^ decreased to 0.3386 ± 0.0324 (mean ± SD, *n* = 6) when region design was removed, indicating that region-aware modeling contributes to improving predictive performance from DNA methylation to gene expression.

Additionally, an experiment on a normal tissue cohort further showed that RSMethy-Net also achieved good predictive performance in noncancer samples (Note S4 and Fig. S4).

In summary, RSMethy-Net consistently outperformed baseline methods across multiple cancer types, with both nonlinear modeling and region-aware design contributing significantly to performance improvement.

### Performance improvement across expression characteristics

**Fig. 3.**
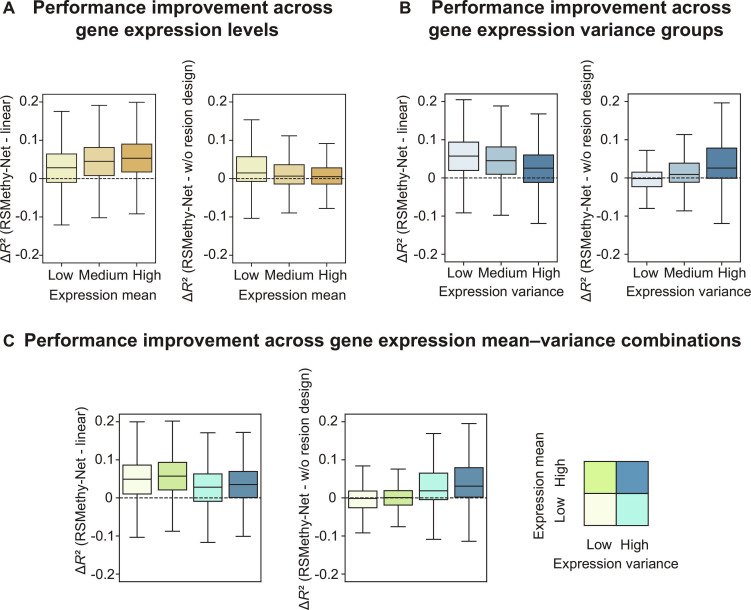
Distribution of performance improvements across different gene expression characteristics. (A) Boxplots of Δ*R*^2^ across gene expression levels (low, medium, and high). (B) Boxplots of Δ*R*^2^ across gene expression variance groups (low, medium, and high). (C) Boxplots of Δ*R*^2^ across combined expression mean–variance groups. Δ*R*^2^ is defined as the difference in gene-level *R*^2^ between RSMethy-Net and 2 reference models: the linear model and the ablation model without region design.

While RSMethy-Net provides consistent predictive improvements across cancer types, we next examined how different genes benefit from the model in terms of performance gains. Genes were grouped according to their expression characteristics, and the performance gain of RSMethy-Net (Δ*R*^2^) relative to 2 reference models (the linear model and the ablation model without region design) was calculated (Fig. 3). Detailed results for each cancer type are provided in Figs. S5 to S10.

First, genes were grouped based on their mean expression levels into low, medium, and high categories using tertile-based grouping, and performance gains were compared with the 2 reference models (Fig. 3A and Figs. S5 and S6). Compared with the linear model (LR), RSMethy-Net showed larger improvements for highly expressed genes, with the high-expression group having the largest median Δ*R*^2^ (0.0530; pairwise Mann–Whitney *U* tests with Bonferroni correction, *P* < 0.001). When compared with the ablation model, the inclusion of region-aware modeling appeared to contribute more to genes with lower expression.

Next, genes were grouped according to expression variance using the same tertile-based grouping strategy (Fig. 3B and Figs. S7 and S8). Relative to the linear model (LR), RSMethy-Net showed relatively stronger gains for genes with lower variance, with the low-variance group showing the highest median Δ*R*^2^ (0.0571; pairwise Mann–Whitney *U* tests with Bonferroni correction, *P* < 0.001), whereas compared with the ablation model, improvements were more apparent for genes with higher variance.

To further examine the combined effect of expression level and variance, genes were additionally grouped using median splits of both mean expression and variance, resulting in 4 mean–variance groups (Fig. 3C and Figs. S9 and S10). The results indicate that, compared with the 2 reference models, RSMethy-Net’s performance gains are more influenced by variance. Specifically, relative to the linear model (LR), low-variance genes showed more improvement overall, with the most pronounced gains observed for genes that are both low-variance and highly expressed. In contrast, relative to the ablation model, improvements were mainly seen for high-variance genes.

These results suggest that RSMethy-Net improves predictive performance across all gene groups, but the magnitude of improvement varies with gene expression characteristics. Nonlinear modeling benefits low-variance genes more, while region-aware modeling contributes relatively more to high-variance genes.

### Characteristics of well-predicted genes

**Fig. 4.**
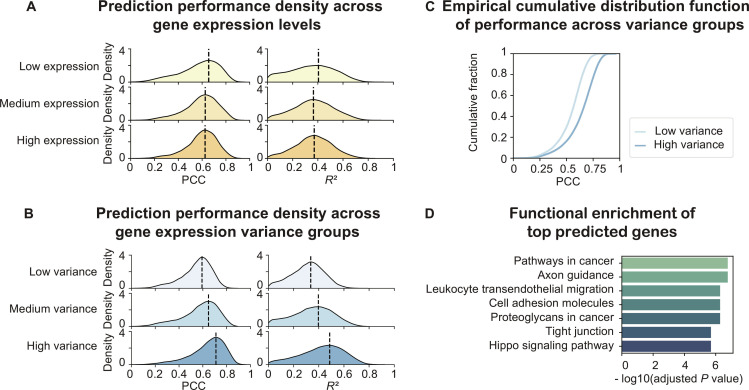
Stratified analysis of prediction performance across gene expression characteristics. (A) Density distributions of prediction performance (*R*^2^ and Pearson correlation coefficient [PCC]) across gene expression levels. (B) Density distributions of prediction performance (*R*^2^ and PCC) across gene expression variance groups. (C) Empirical cumulative distribution function of PCC for high- and low-variance genes, illustrating differences in prediction performance between the 2 groups. (D) Functional enrichment analysis of the top 10% genes with the highest prediction performance.

To further understand which types of genes are better predicted, we performed a stratified analysis of RSMethy-Net’s performance across all predicted genes and examined the relationship between gene expression characteristics and prediction accuracy (Fig. 4).

Genes were stratified into tertiles based on mean expression level and expression variance, and the distributions of predictive performance within each group were compared (Fig. 4A and B). The results showed that RSMethy-Net’s performance distributions were generally similar across different expression levels, suggesting that mean expression has a relatively limited effect on prediction accuracy (Fig. 4A). In contrast, expression variance showed a clearer relationship with predictive performance, with high-variance genes generally predicted more accurately (Fig. 4B). Detailed results for each cancer type are provided in Figs. S11 to S14.

This trend was further supported by the empirical cumulative distribution function of PCC (Fig. 4C), showing that high-variance genes tended to have PCC values shifted toward higher values.

Additionally, to explore the potential biological characteristics of well-predicted genes, the top 10% of genes ranked by predictive performance were subjected to KEGG pathway enrichment analysis [46] (Fig. 4D). These well-predicted genes were enriched in multiple pathways related to cancer progression, including Pathways in cancer, Cell adhesion molecules, and Proteoglycans in cancer, which involve critical cancer-related processes such as cell proliferation, migration, and tumor invasion. In contrast, when performing the same analysis in normal tissues (Solid Tissue Normal), no pathways remained significant after multiple-testing correction.

Overall, genes with higher expression variance tend to be better predicted by RSMethy-Net. In cancer cohorts, well-predicted genes are enriched in cancer-related pathways, while such signals were not consistently observed in normal tissues, suggesting potential context-specific patterns in the model’s predictive behavior.

### Model interpretability and regional contributions

The structured framework of RSMethy-Net provides a solid foundation for model interpretability. We quantified the contribution of different gene regions to expression prediction using the model’s learned relationships, providing a quantitative analysis of regional attribution for predictions.

In previous DNA methylation multi-omics studies, correlations between methylation levels in different gene regions and gene expression are often used to characterize region-level regulatory relationships. For example, methylation near TSS is generally negatively associated with expression [19–21], whereas gene body methylation tends to be positively associated [22–24]. Traditional correlation analyses primarily capture linear relationships. In contrast, RSMethy-Net employs a region-aware design, where region encoders are used to model nonlinear relationships between methylation signals and gene expression across genomic regions. Consequently, the model’s utilization of different regional features may reflect more complex, nonlinear associations.

**Fig. 5.**
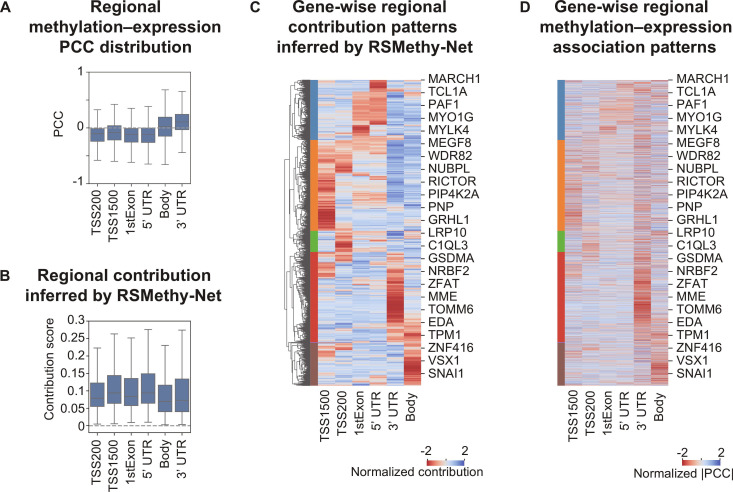
Regional contributions revealed by interpretability analysis of RSMethy-Net. (A) Distribution of PCC between DNA methylation levels in different genomic regions and gene expression. (B) Distribution of regional contribution scores inferred by RSMethy-Net, obtained from the model interpretability analysis in each genomic region. (C) Clustered heatmap showing the normalized regional contribution for each gene across genomic regions, representing gene-wise regional contribution patterns revealed by the interpretability analysis of RSMethy-Net. (D) Heatmap of normalized absolute PCC values between regional methylation and gene expression. Genes are ordered according to the clustering structure obtained in (C), enabling direct comparison between RSMethy-Net-derived regional contributions and PCC-based association patterns.

For example, using the LUAD dataset, which has a relatively large sample size, we applied gradient-based saliency methods [44,45] to quantify the contribution of each gene region and compared the results with traditional correlation analyses (Fig. 5). First, using conventional methods, correlations between methylation levels in different regions and gene expression were calculated (Fig. 5A). The results showed that methylation in TSS200, TSS1500, 1stExon, and 5′UTR regions was generally negatively correlated with expression, whereas methylation in Body and 3′UTR regions showed positive trends. The absolute PCC values were generally low, and this trend is consistent with conventional understanding. Using model interpretability analysis, we obtained distributions of regional contribution scores (Fig. 5B). The resulting contribution patterns showed partial consistency with trends observed in PCC-based correlation analyses.

We additionally performed region-level contribution analyses for representative genes in LUAD using epidermal growth factor receptor (EGFR) and phosphatase and tensin homolog (PTEN) as case studies (Fig. S15). The results show that EGFR exhibits higher contributions in promoter-proximal regions (TSS), which is consistent with the known relevance of promoter regions in transcriptional regulation. For PTEN, relatively higher contributions are observed in the 3′UTR and gene body regions. Previous studies have reported that PTEN is involved in posttranscriptional regulation through miRNA-mediated interactions and ceRNA-related networks [47], where miRNA binding sites are commonly enriched within 3′UTR regions. These results are consistent with model-derived attributions and existing biological knowledge.

To further understand the relationship between model-inferred regional contribution scores and traditional correlation analyses, we clustered the contribution scores across different regions for each gene. Figure 5C presents a heatmap of the resulting regional contribution patterns derived from RSMethy-Net. Distinct genes exhibited clearly different distributions of regional contributions, giving rise to several gene clusters with distinct regional characteristics. For comparison, the absolute PCC values between methylation levels and gene expression for each gene region were calculated and visualized in a corresponding heatmap (Fig. 5D), with genes ordered according to the clustering shown in Fig. 5C. This setup allows a direct comparison between the model-inferred regional contribution patterns and correlation-based patterns. The results indicate that the regional patterns obtained from the 2 methods were broadly consistent overall, such that the cluster structures observed in Fig. 5C were reflected in similar trends in Fig. 5D, yet some differences remained.

These results indicate that, in the task of predicting transcriptomic expression from DNA methylation, RSMethy-Net’s interpretability analysis can reveal the contributions of different regions to the predictions. Genes exhibit diverse regional contribution patterns, which are broadly consistent with traditional methylation–expression correlation trends, while also providing additional information insight by the nonlinear modeling framework.

## Discussion and Conclusion

In this study, we proposed a region-aware encoding-based computational framework, RSMethy-Net, for predicting gene expression levels from DNA methylation data. Through systematic evaluation on 6 TCGA cancer cohorts, we found that the model consistently outperformed multiple baseline methods across various metrics. Ablation experiments further indicated that both the nonlinear modeling capability and the structured region design based on genomic regions played important roles in improving predictive performance. We also explored alternative approaches for incorporating region information, such as adding region summary features. The results indicated that the region-aware encoder provides a more effective and suitable modeling strategy (Table S5). Beyond predictive accuracy, this task is motivated by the need to better understand regulatory relationships between DNA methylation and gene expression. From a biological perspective, DNA methylation is an upstream epigenetic modification associated with transcriptional regulation, motivating the modeling of methylation-to-expression relationships as a biologically grounded predictive task. Although gene expression data are often available in practice, methylation-based modeling provides a complementary framework for analyzing region-aware and nonlinear epigenetic regulation underlying transcription.

Further group-based analyses in the “Performance improvement across expression characteristics” section revealed that the model’s performance gains varied across different types of genes, suggesting that gene expression characteristics are closely associated with differences in methylation–expression regulatory complexity. Compared with linear models, RSMethy-Net showed more noticeable improvements for low-variance genes, which may be attributed to the potentially more nonlinear methylation–expression regulatory patterns at low-variance gene levels. In contrast, when compared with the ablation model, the region-aware modeling structure contributed more substantially to performance gains in high-variance genes. This may indicate that different genomic regions could play more complex and cooperative roles in the regulation of highly variable genes. Although the overall network size remained approximately the same, a single encoder might be insufficient to capture the heterogeneity among multiple regional signals, whereas region-aware encoding strategies could more effectively capture such structured regulatory patterns, potentially contributing to the observed performance improvements.

Model interpretability analyses further highlighted the regional contribution patterns that different genes relied on during prediction. Overall, these regional contributions generally aligned with trends observed in conventional methylation–gene expression correlation analyses, while also showing some tendencies that differed from linear correlations. We speculate that the region contributions obtained through model interpretability may reflect the degree of association between methylation and gene expression under a nonlinear framework.

Despite these advances, several limitations remain. While the model shows good performance in within-cancer validation across TCGA cancer cohorts, its performance under cross-cancer settings is relatively limited (Note S5 and Fig. S16). The current study only utilizes DNA methylation information for prediction, whereas gene expression regulation is influenced by multiple epigenetic factors. Future work will explore how to incorporate regional functional information across additional omics data to develop more accurate and comprehensive computational omics approaches. Moreover, the current interpretability analyses are primarily computational and show trends that are generally consistent with conventional correlation analyses, but they lack experimental validation. Further wet-lab studies would be valuable to confirm and extend these findings.

Overall, the RSMethy-Net computational framework proposed in this study effectively improves predictive performance for DNA methylation-based transcriptome prediction tasks by incorporating region-aware encoding strategies combined with nonlinear modeling and provides a new perspective for understanding the potential roles of different gene regions in methylation–transcriptional regulation.

## Data Availability

The code is available at https://github.com/Fuu-w/RSMethy-Net. All data used in this study were previously published and publicly available. DNA methylation and gene expression data were downloaded from the UCSC Xena [30], which hosts datasets from TCGA [29].

## Supplementary Materials

Supplementary 1 Notes S1 to S5 Figs. S1 to S16 Tables S1 to S5
