# Supplementary material for: A Region-Aware Structured Framework Improves Prediction of Gene Expression from DNA Methylation
Source: Comput Struct Biotechnol J. 2026 Jun 26;35(1):0138. doi: 10.34133/csbj.0138 (PMC13305029; doi:10.34133/csbj.0138)
Supplement: Supplementary 1 — Notes S1 to S5 Figs. S1 to S16 Tables S1 to S5 [file csbj.0138.f1.pdf]

Supplementary Information for

A Region-Aware Structured Framework Improves Prediction of Gene Expression from DNA Methylation

Zhixing Zhong[1], Jinglu Hu*[1]

[1] Graduate School of Information, Production and Systems, Waseda University, Fukuoka, 808-0135, Japan

Corresponding author: jinglu@waseda.jp (Jinglu Hu)

5 Supplementary Notes

16 Supplementary Figures

5 Supplementary Tables

Supplementary Note S1. Selection of preprocessing parameters

The gene expression filtering threshold and CpG selection window size were selected following commonly adopted settings in previous DNA methylation-based gene expression prediction studies. Additional sensitivity analyses showed substantially reduced predictive performance for genes with average expression < 1 (**Supplementary Figure S1**). Sensitivity analyses across different CpG selection window sizes further showed that larger windows did not provide clear performance improvements but increased computational cost, supporting the use of the ±10 Mb window (**Supplementary Figure S2**).

Supplementary Note S2. Evaluation of grouped-region and region-specific encoder architectures

To incorporate genomic regions with similar regulatory tendencies into a shared latent representation, the grouped region encoder was adopted in the main model architecture. To further evaluate the impact of this regional grouping strategy, we also assessed the region-specific encoder architecture, in which each genomic region was modeled independently, and compared its performance with that of the grouped-region encoder across datasets using R^2, PCC, MSE, and MAE under five-fold patient-level cross-validation. The results are shown in **Supplementary Table S2**. The grouped-region encoder consistently achieved improved predictive performance, supporting the effectiveness and rationality of modeling functionally related genomic regions jointly.

Supplementary Note S3. Detailed Architecture and Dimensional Settings of RSMethy-Net

To clarify the architectural design and parameter configuration of RSMethy-Net, we provide additional implementation details here.

We denote n_m as the number of functional regions with available methylation probe coverage in region group G_m for a given gene. Due to variability in CpG probe annotation across genes and datasets, n_m may differ across genes. However, under complete regional coverage, the maximum number of functional regions is $n_1 = 4$ for G_1 and $n_2 = 2$ for G_2. In practice, the actual values of n_m may be smaller depending on probe availability. For the "others" group, we set $n_3 = 1$, representing a single aggregated region. For each gene-specific model, n_m is fixed once CpG-to-region mapping is determined prior to training, ensuring consistent dimensionality during training and inference.

Each grouped encoder adopts a two-layer fully connected architecture (Linear → ReLU → Linear).

We denote d_m as the number of CpG probes assigned to group G_m for a given gene. Since CpG probe coverage differs across genes, d_m is gene-dependent. However, each gene is modeled independently using a separately trained gene-specific neural network, ensuring a fixed input dimension within each model during training and inference.

CpG-to-gene mapping is determined prior to model training and remains fixed throughout training and inference. Each gene-specific model operates in its own fixed input space defined by its associated CpG set. Therefore, differences in CpG counts across genes do not affect model consistency, as each model is trained independently.

For each group G_m, the grouped encoder outputs a representation with dimensionality $n_m \cdot 64$ (i.e., $k = 64$).

Representations from all groups are concatenated to form a vector of dimension $(n_1 + n_2 + n_3) \cdot 64$, which is then fed into the predictor module. The predictor consists of a two-layer fully connected network (Linear → ReLU → Linear) and outputs the final gene expression value.

Supplementary Note S4. Performance Evaluation on Normal Tissue Cohort.

We constructed a normal tissue cohort by integrating samples labeled as “11” (Solid Tissue Normal) from six TCGA cancer types, resulting in 88 samples. Additional experiments using cross-validation were performed on this cohort to evaluate model performance under non-cancer conditions, and the results are shown in **Supplementary Figure S4**. RSMethy-Net showed good performance on normal tissues, with generally better predictive performance compared with cancer cohorts.

Supplementary Note S5. Cross-cancer-type generalization

External validation across independent datasets is limited by the availability of matched DNA methylation and gene expression data. To partially address this, we performed a cross-cancer-type evaluation by training on multiple cancer types and testing on a held-out cancer type not used during training, without retraining or fine-tuning.

The results show decreased R^2 compared with within-cohort validation, while PCC remains at a moderate level (**Supplementary Figure S16**). This suggests reduced accuracy in predicting absolute expression across cancer types, but relatively stable preservation of rank-based expression patterns.

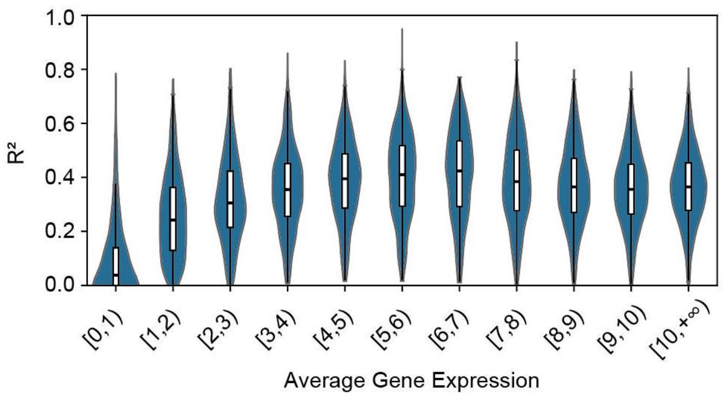

Supplementary Figure S1. Sensitivity analysis of prediction performance across genes with different average expression levels in the LUAD dataset.

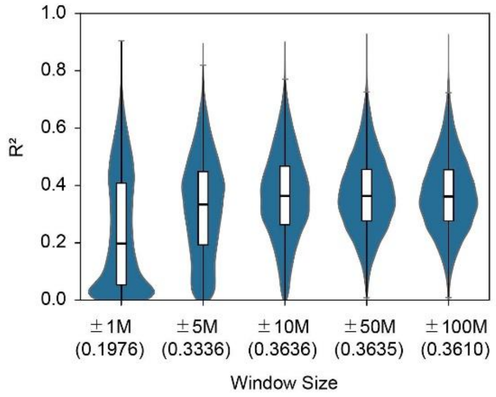

Supplementary Figure S2. Sensitivity analysis of model performance under different CpG selection window sizes in the LUAD dataset. The numbers below each window size on the x-axis indicate the median performance values.

p < 0.001	p < 0.001	p < 0.001	p < 0.001	p < 0.001	HNSC
p < 0.001	p < 0.001	p < 0.001	p < 0.001	p < 0.001	LUAD
p < 0.001	p < 0.001	p < 0.001	p < 0.001	p < 0.001	STAD
p < 0.001	p < 0.001	p < 0.001	p < 0.001	p < 0.001	COAD
p < 0.001	p < 0.001	p < 0.001	p < 0.001	p < 0.001	UCEC
p < 0.01	p < 0.001	p < 0.001	p < 0.001	p < 0.001	PAAD

RSMethy-Net without Region Design
Linear Regression
Random Forest
Support Vector Machine
Convolutional Neural Networks

Supplementary Figure S3. Statistical comparison of R^2 between RSMethy-Net and competing methods using paired Wilcoxon signed-rank tests across genes within each cancer type. Reported p-values are Bonferroni-adjusted.

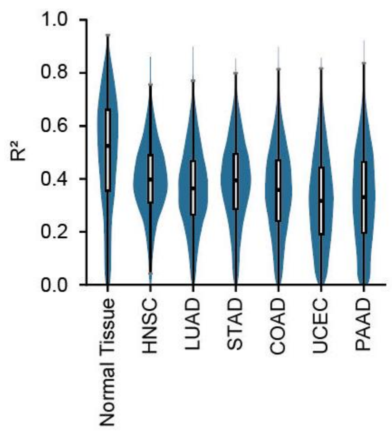

Supplementary Figure S4. RSMethy-Net performance in normal tissue and cancer cohorts.

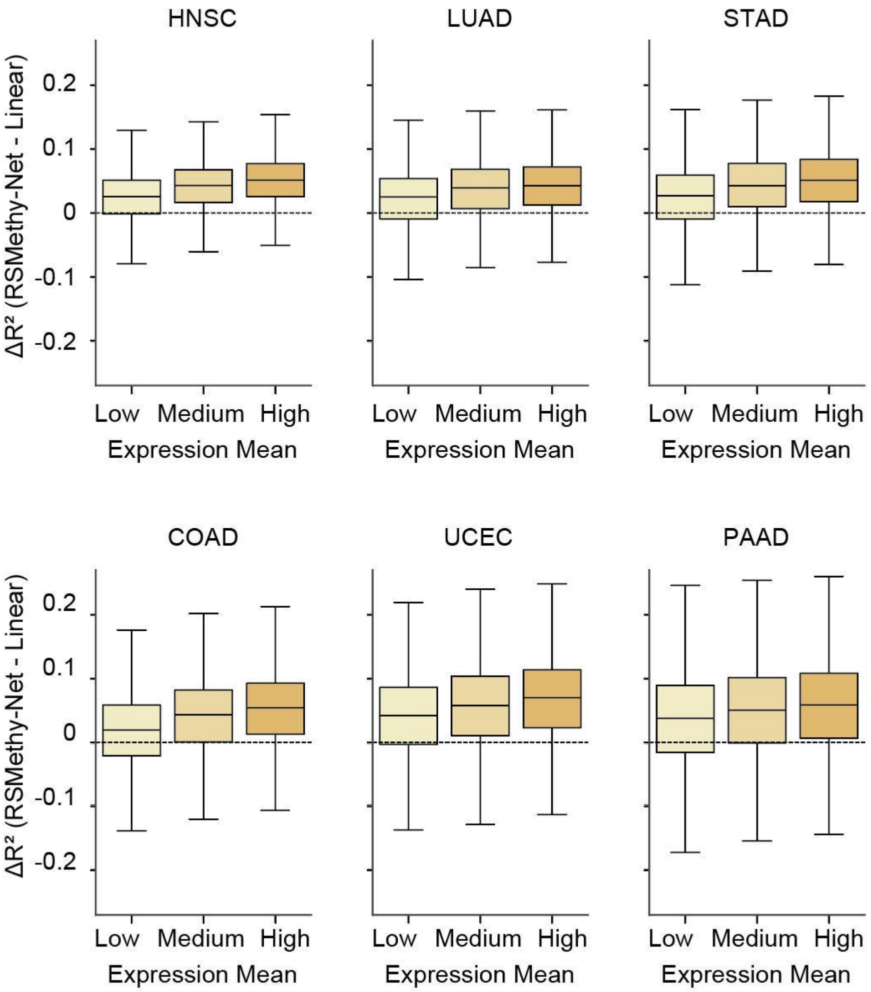

Supplementary Figure S5. Distribution of performance improvements across different gene expression levels relative to the linear model. ΔR^2 is defined as the difference in gene-level R^2 between RSMethy-Net and the linear model.

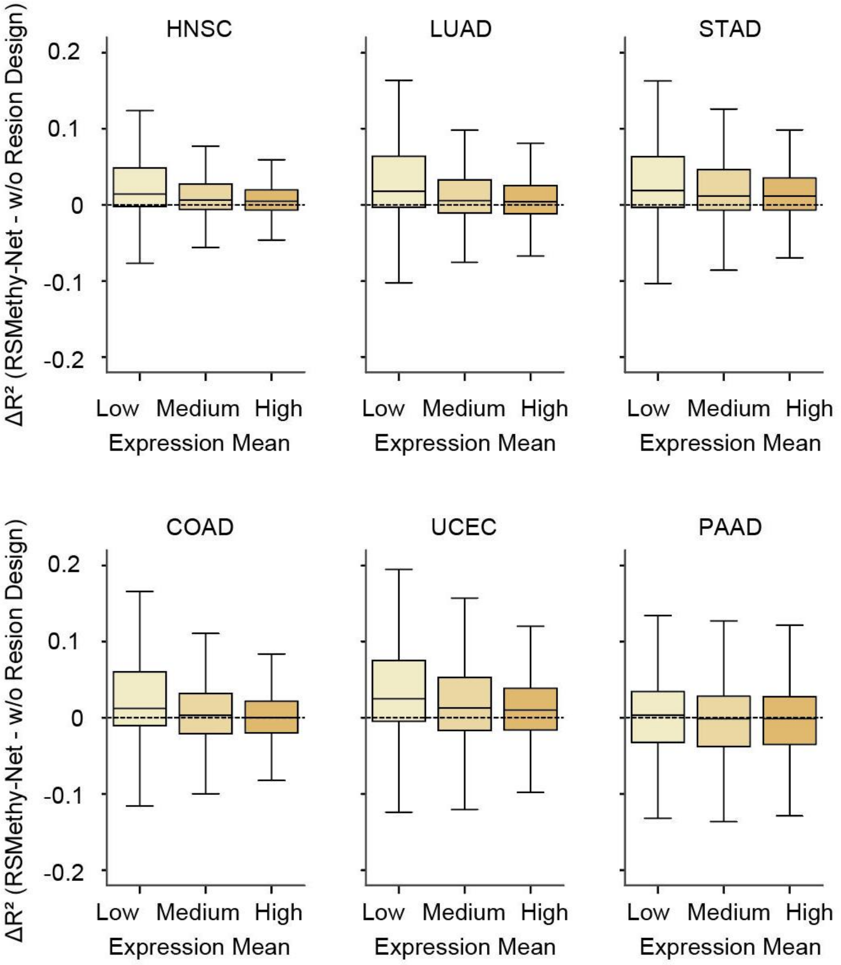

Supplementary Figure S6. Distribution of performance improvements across different gene expression levels relative to the ablation model without region design. ΔR^2 is defined as the difference in gene-level R^2 between RSMethy-Net and the ablation model without region design.

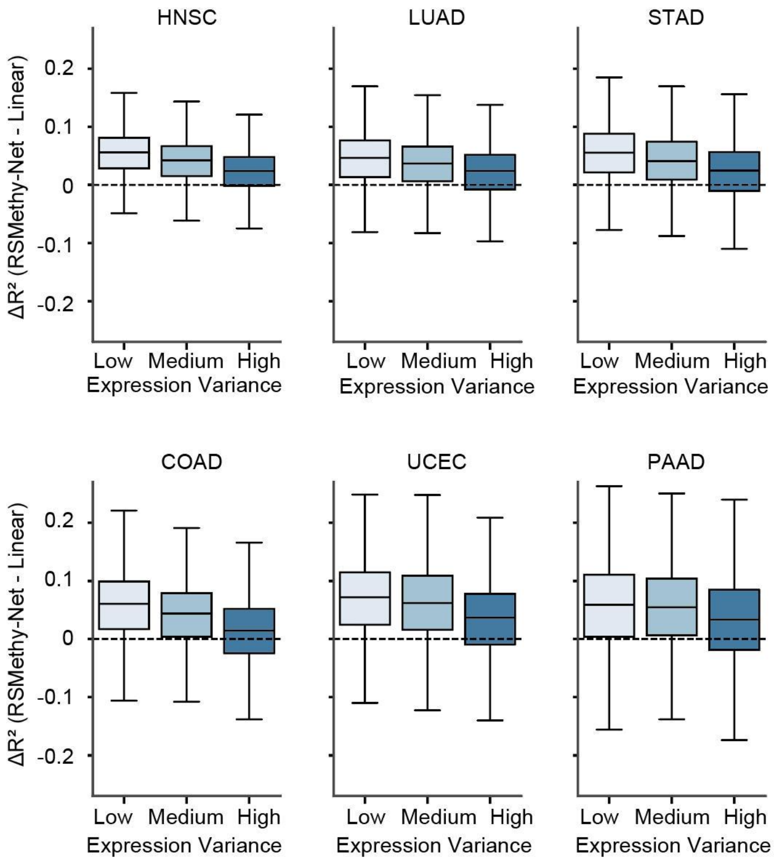

Supplementary Figure S7. Distribution of performance improvements across different gene expression variance groups relative to the linear model. ΔR^2 is defined as the difference in gene-level R^2 between RSMethy-Net and the linear model.

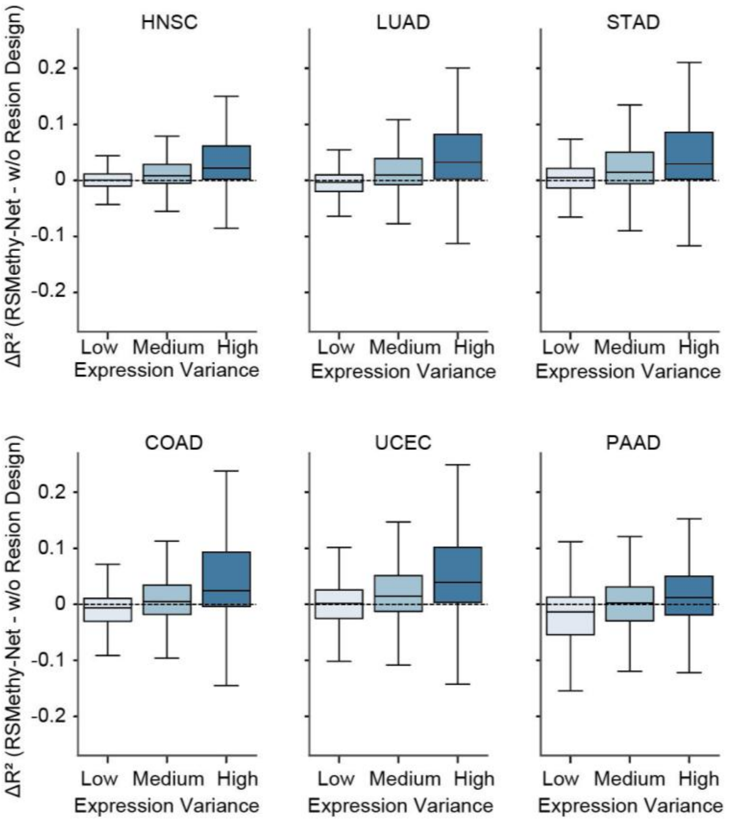

Supplementary Figure S8. Distribution of performance improvements across different gene expression variance groups relative to the ablation model without region design. ΔR^2 is defined as the difference in gene-level R^2 between RSMethy-Net and the ablation model without region design.

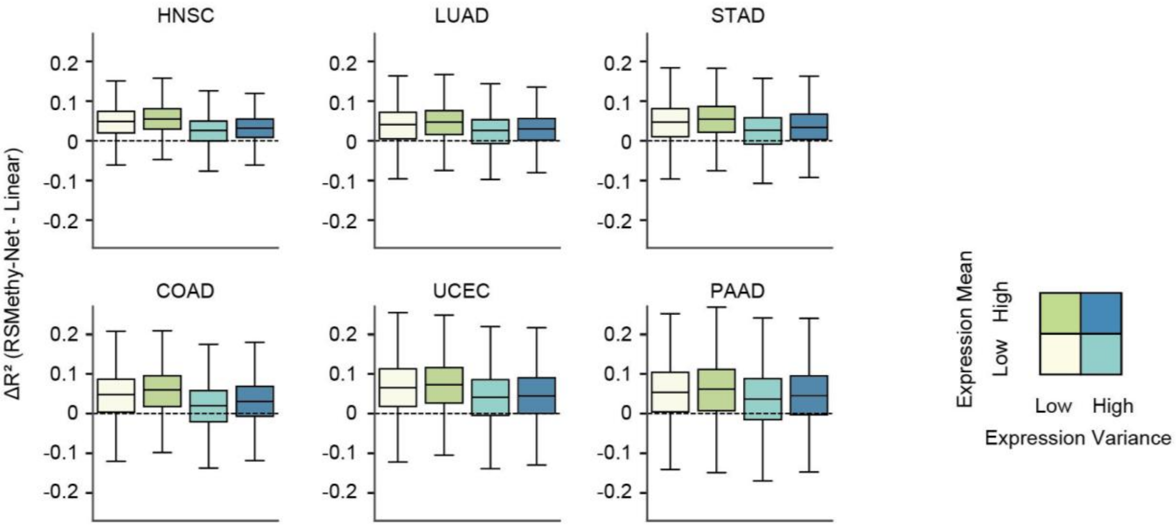

Supplementary Figure S9. Distribution of performance improvements across combined expression mean–variance groups relative to the linear model. ΔR^2 is defined as the difference in gene-level R^2 between RSMethy-Net and the linear model.

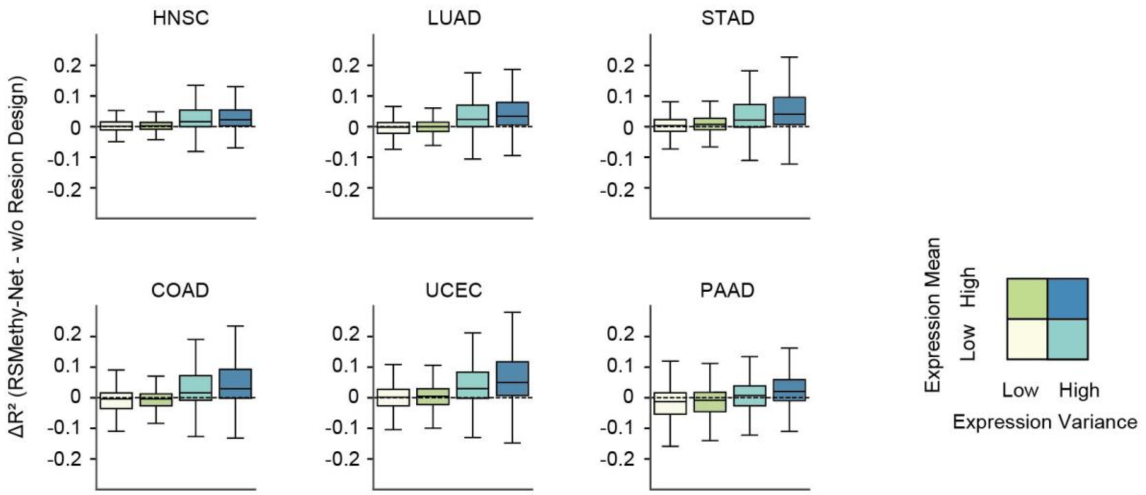

Supplementary Figure S10. Distribution of performance improvements across combined expression mean–variance groups relative to the ablation model without region design. ΔR^2 is defined as the difference in gene-level R^2 between RSMethy-Net and the ablation model without region design.

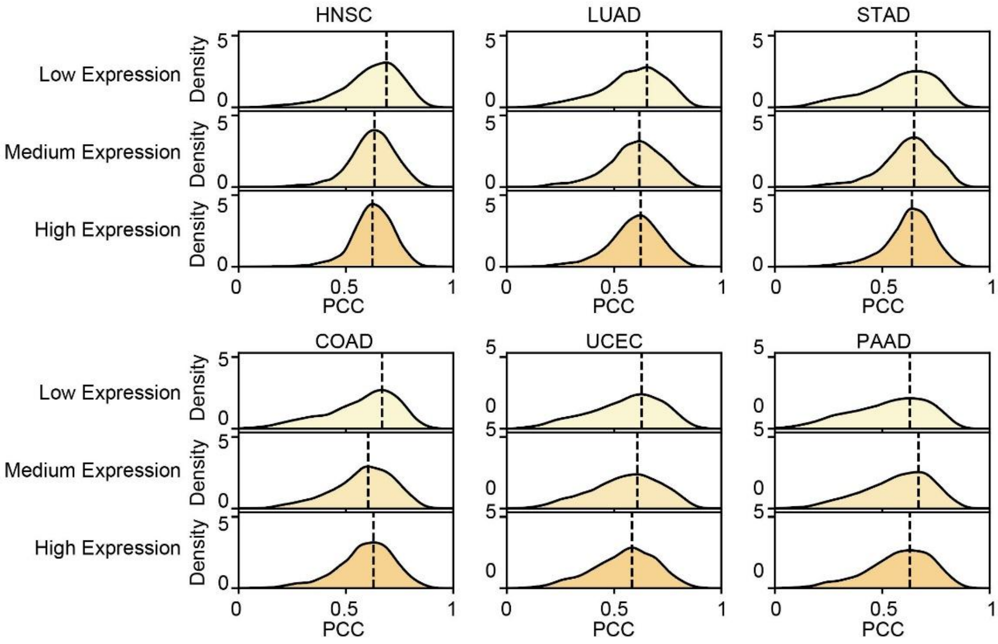

Supplementary Figure S11. Density distributions of PCC values across gene expression levels.

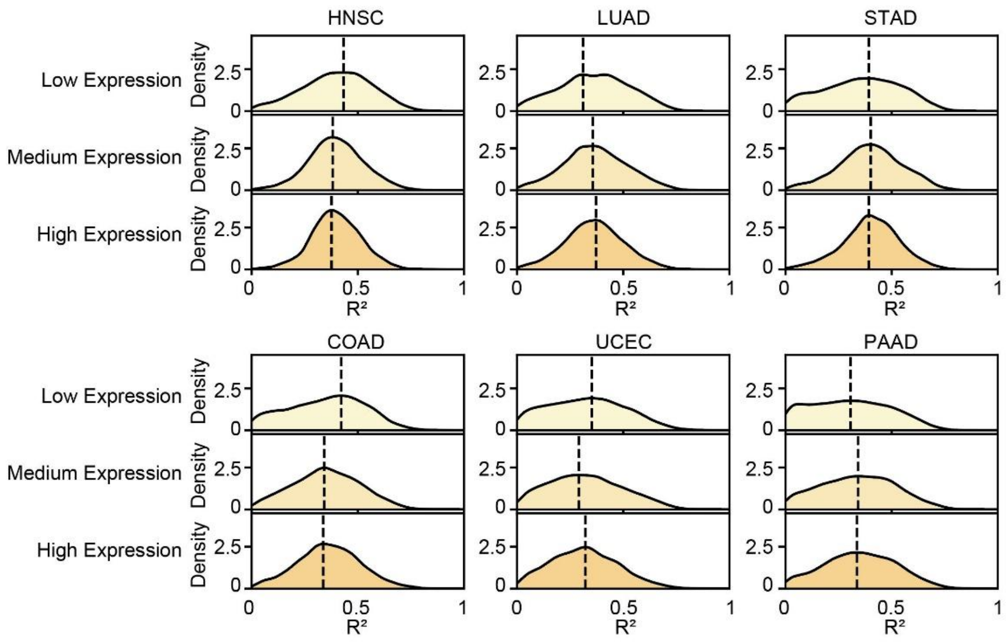

Supplementary Figure S12. Density distributions of R^2 values across gene expression levels.

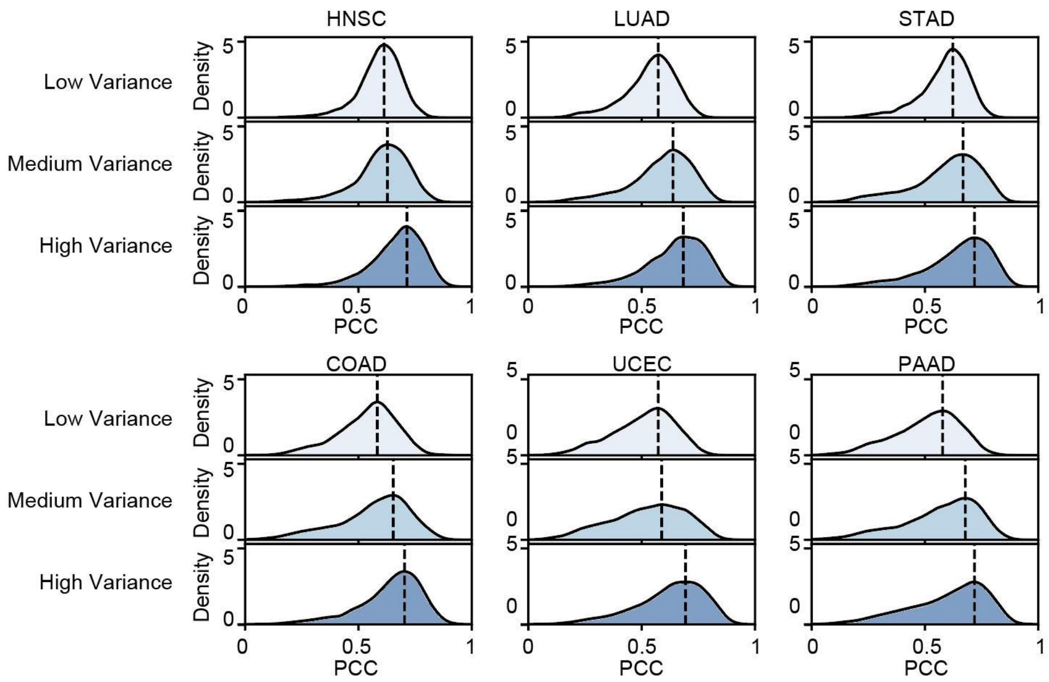

Supplementary Figure S13. Density distributions of PCC values across gene expression variance groups.

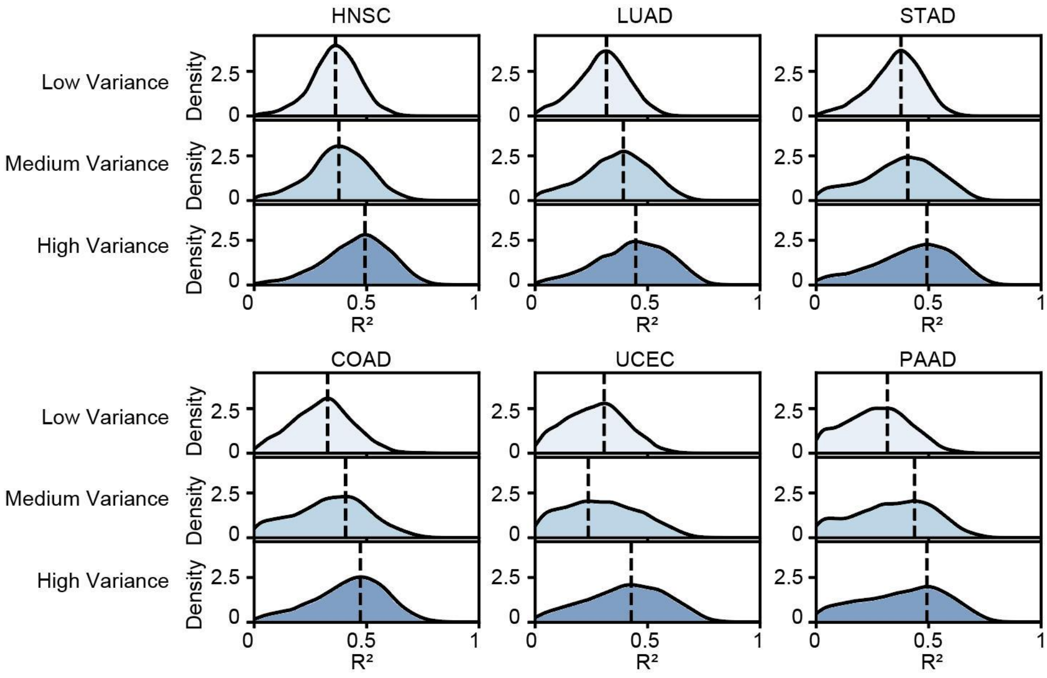

Supplementary Figure S14. Density distributions of R^2 values across gene expression variance groups.

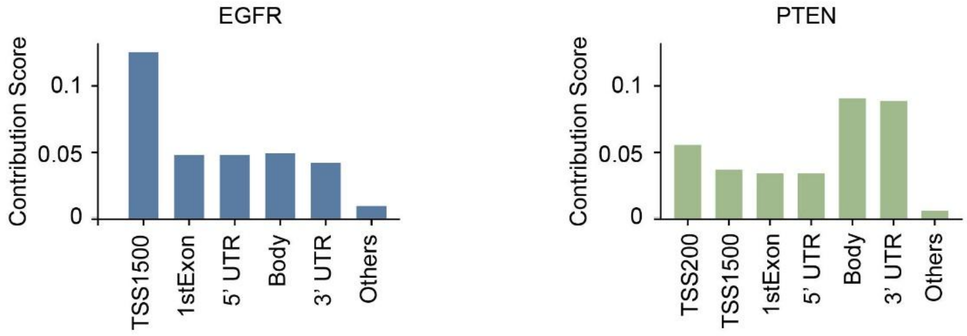

Supplementary Figure S15. Region-level contribution analysis for representative genes in LUAD. Gradient-based attributions are aggregated across genomic regions to assess model interpretability at the gene level. The figure illustrates EGFR and PTEN, with no probes mapped to the TSS200 region of EGFR.

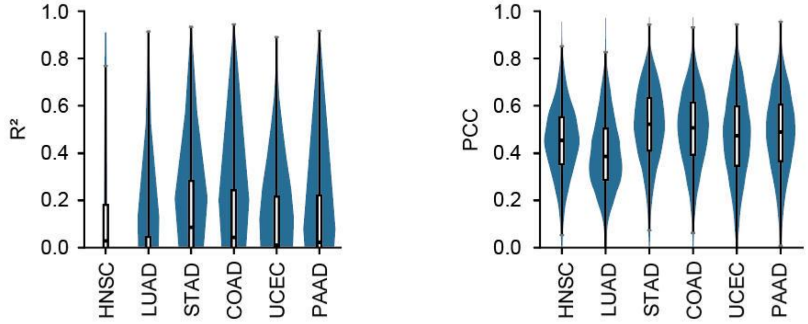

Supplementary Figure S16. Cross-cancer-type validation performance (R^2 and PCC).

Supplementary Table S1. Numbers of samples and genes included for each cancer cohort after preprocessing and filtering.

Cancer Type	Samples	Genes
HNSC	542	14,036
LUAD	477	14,148
STAD	372	14,286
COAD	299	13,826
UCEC	197	14,155
PAAD	183	14,279

Supplementary Table S2. Performance comparison about the grouped-region and the region-specific encoder architectures. Median gene-level R^2, PCC, MSE and MAE under five-fold patient-level cross-validation are reported for each cancer type.

Cancer Type	Method	Performance			
		**Median R^2**	**Median PCC**	**Median MSE**	**Median MAE**
HNSC	No Region Design	0.3798	0.6181	0.4468	0.5041
	Region-specific Encoder	0.3915	0.6280	0.4405	0.5028
	Grouped-region Encoder	0.3969	0.6321	0.4322	0.4977
LUAD	No Region Design	0.3400	0.5848	0.4172	0.4888
	Region-specific Encoder	0.3538	0.5975	0.4128	0.4871
	Grouped-region Encoder	0.3636	0.6057	0.4011	0.4812
STAD	No Region Design	0.3624	0.6039	0.4297	0.4986
	Region-specific Encoder	0.3828	0.6217	0.4221	0.4965
	Grouped-region Encoder	0.3936	0.6299	0.4085	0.4893
COAD	No Region Design	0.3341	0.5811	0.3035	0.4198
	Region-specific Encoder	0.3489	0.5953	0.3034	0.4215
	Grouped-region Encoder	0.3586	0.6023	0.2953	0.4166
UCEC	No Region Design	0.2848	0.5387	0.6730	0.6090
	Region-specific Encoder	0.3034	0.5574	0.6574	0.6060
	Grouped-region Encoder	0.3172	0.5695	0.6389	0.5965
PAAD	No Region Design	0.3307	0.5825	0.323	0.4200
	Region-specific Encoder	0.3316	0.5841	0.3222	0.4204
	Grouped-region Encoder	0.3313	0.5837	0.3212	0.4194

Supplementary Table S3. Mean and standard deviation of predictive performance for six methods across six TCGA cancer cohorts. Gene-level R^2 and PCC under five-fold patient-level cross-validation are reported as mean and standard deviation (SD) for each cancer type.

Cancer Type	Method	Performance			
		**Mean R^2**	**SD R^2**	**Mean PCC**	**SD PCC**
HNSC	Convolutional Neural Networks	0.2957	0.1465	0.5321	0.1335
	Support Vector Machine	0.3266	0.1245	0.5742	0.1060
	Random Forest	0.2699	0.1066	0.5414	0.1119
	Linear Regression	0.3604	0.1394	0.5894	0.1221
	RSMethy-Net without Region Design	0.3797	0.1224	0.6096	0.1046
	RSMethy-Net (ours)	0.3975	0.1369	0.6221	0.1163
LUAD	Convolutional Neural Networks	0.2730	0.1501	0.5074	0.1431
	Support Vector Machine	0.2905	0.1288	0.5421	0.1147
	Random Forest	0.2348	0.1126	0.4995	0.1246
	Linear Regression	0.3343	0.1491	0.5643	0.1354
	RSMethy-Net without Region Design	0.3452	0.1279	0.5792	0.1136
	RSMethy-Net (ours)	0.3643	0.1513	0.5917	0.1345
STAD	Convolutional Neural Networks	0.2894	0.1532	0.5241	0.1425
	Support Vector Machine	0.2992	0.1427	0.5503	0.1262
	Random Forest	0.2660	0.1280	0.5203	0.1339
	Linear Regression	0.3496	0.1554	0.5762	0.1428
	RSMethy-Net without Region Design	0.3601	0.1395	0.5900	0.1245
	RSMethy -Net (ours)	0.3861	0.1563	0.6094	0.1378
COAD	Convolutional Neural Networks	0.2415	0.1777	0.4845	0.1608
	Support Vector Machine	0.2819	0.1435	0.5381	0.1307
	Random Forest	0.2495	0.1309	0.5055	0.1418
	Linear Regression	0.3198	0.1659	0.5465	0.1595
	RSMethy-Net without Region Design	0.3372	0.1403	0.5693	0.1300
	RSMethy-Net (ours)	0.3544	0.1618	0.5806	0.1498
UCEC	Convolutional Neural Networks	0.2476	0.1763	0.4868	0.1624
	Support Vector Machine	0.2410	0.1505	0.5025	0.1416
	Random Forest	0.2289	0.1382	0.4756	0.1579
	Linear Regression	0.2676	0.1822	0.4909	0.1899
	RSMethy-Net without Region Design	0.2941	0.1449	0.5297	0.1407
	RSMethy-Net (ours)	0.3218	0.1701	0.5514	0.1601
PAAD	Convolutional Neural Networks	0.2546	0.2019	0.4982	0.1740
	Support Vector Machine	0.2254	0.1407	0.5232	0.1447
	Random Forest	0.2604	0.1420	0.5124	0.1607
	Linear Regression	0.2855	0.1780	0.5113	0.1822
	RSMethy-Net without Region Design	0.3341	0.1559	0.5658	0.1490
	RSMethy-Net (ours)	0.3308	0.1765	0.5600	0.1654

Supplementary Table S4. Mean ± standard deviation of median predictive performance across six TCGA cancer cohorts.

Method	R^2	PCC	MSE	MAE
Convolutional Neural Networks	0.2524 ± 0.0208	0.5073 ± 0.0186	0.4726 ± 0.1299	0.5209 ± 0.0688
Support Vector Machine	0.2735 ± 0.0392	0.5461 ± 0.0237	0.4758 ± 0.1369	0.5214 ± 0.0692
Random Forest	0.2351 ± 0.0200	0.5133 ± 0.0224	0.4965 ± 0.1383	0.5321 ± 0.0730
Linear Regression	0.3092 ± 0.0395	0.5577 ± 0.0346	0.4448 ± 0.1327	0.5055 ± 0.0715
RSMethy-Net without Region Design	0.3386 ± 0.0324	0.5848 ± 0.0269	0.4322 ± 0.1319	0.4901 ± 0.0697
RSMethy-Net (ours)	0.3602 ± 0.0321	0.6039 ± 0.0247	0.4162 ± 0.1215	0.4835 ± 0.0657

Supplementary Table S5. Performance Comparison of Models with and without Region Summary Features and Region Encoders. Median gene-level R^2, PCC, MSE and MAE under five-fold patient-level cross-validation are reported for each cancer type.

Cancer Type	Method	Performance			
		Median R^2	Median PCC	Median MSE	Median MAE
HNSC	SVM	0.3255	0.5811	0.4816	0.5306
	SVM + Region Summary	0.3277	0.5826	0.4799	0.5297
	RF	0.2578	0.5429	0.5307	0.5569
	RF + Region Summary	0.2599	0.5455	0.5287	0.5564
	LR	0.3518	0.5946	0.4610	0.5192
	LR + Region Summary	0.3520	0.5948	0.4605	0.5192
	Ablation (No Region Design)	0.3798	0.6181	0.4468	0.5041
	Ablation + Region Summary	0.3824	0.6202	0.4446	0.5033
	RSMethy-Net	0.3969	0.6321	0.4322	0.4977
LUAD	SVM	0.2852	0.5458	0.4555	0.5155
	SVM + Region Summary	0.2880	0.5487	0.4530	0.5147
	RF	0.2175	0.4989	0.4949	0.5357
	RF + Region Summary	0.2197	0.5020	0.4923	0.5348
	LR	0.3224	0.5691	0.4216	0.4978
	LR + Region Summary	0.3228	0.5694	0.4215	0.4978
	Ablation (No Region Design)	0.3400	0.5848	0.4172	0.4888
	Ablation + Region Summary	0.3422	0.5870	0.4170	0.4882
	RSMethy-Net	0.3636	0.6057	0.4011	0.4812
STAD	SVM	0.2978	0.5597	0.4751	0.5300
	SVM + Region Summary	0.3014	0.5622	0.4729	0.5292
	RF	0.2514	0.5269	0.5034	0.5462
	RF + Region Summary	0.2536	0.5291	0.5010	0.5458
	LR	0.3453	0.5894	0.4337	0.5093
	LR + Region Summary	0.3456	0.5898	0.4329	0.5092
	Ablation (No Region Design)	0.3624	0.6039	0.4297	0.4986
	Ablation + Region Summary	0.3639	0.6055	0.4288	0.4986
	RSMethy-Net	0.3936	0.6299	0.4085	0.4893
COAD	SVM	0.2770	0.5453	0.3306	0.4434
	SVM + Region Summary	0.2802	0.5478	0.3298	0.4424
	RF	0.2295	0.5090	0.3480	0.4543
	RF + Region Summary	0.2315	0.5115	0.3475	0.4543
	LR	0.3089	0.5582	0.3142	0.4336
	LR + Region Summary	0.3096	0.5585	0.3140	0.4338
	Ablation (No Region Design)	0.3341	0.5811	0.3035	0.4198
	Ablation + Region Summary	0.3361	0.5832	0.3031	0.4188
	RSMethy-Net	0.3586	0.6023	0.2953	0.4166
UCEC	SVM	0.2338	0.5103	0.7282	0.6424
	SVM + Region Summary	0.2371	0.5131	0.7259	0.6419

	RF	0.2076	0.4796	0.7333	0.6476
	RF + Region Summary	0.2101	0.4832	0.7315	0.6461
	LR	0.2511	0.5061	0.6910	0.6321
	LR + Region Summary	0.2514	0.5066	0.6906	0.6319
	Ablation (No Region Design)	0.2848	0.5387	0.6730	0.6090
	Ablation + Region Summary	0.2878	0.5420	0.6716	0.6070
	RSMethy-Net	0.3172	0.5695	0.6389	0.5965
PAAD	SVM	0.2219	0.5346	0.3837	0.4667
	SVM + Region Summary	0.2229	0.5352	0.3835	0.4666
	RF	0.2464	0.5223	0.3690	0.4521
	RF + Region Summary	0.2465	0.5229	0.3686	0.4527
	LR	0.2754	0.5290	0.3473	0.4411
	LR + Region Summary	0.2760	0.5293	0.3464	0.4407
	Ablation (No Region Design)	0.3307	0.5825	0.3230	0.4200
	Ablation + Region Summary	0.3321	0.5835	0.3229	0.4198
	RSMethy-Net	0.3313	0.5837	0.3212	0.4194
